# Comparison between remote sensing and a dynamic vegetation model for estimating terrestrial primary production of Africa

**DOI:** 10.1186/s13021-015-0018-5

**Published:** 2015-03-31

**Authors:** Jonas Ardö

**Affiliations:** Department of Physical Geography and Ecosystem Science, Lund University, Sölvegatan 12, Lund, 223-62 Sweden

**Keywords:** Africa, MOD17, NPP, GPP, LPJ-GUESS, Resource assessment, Carbon cycle

## Abstract

**Background:**

Africa is an important part of the global carbon cycle. It is also a continent facing potential problems due to increasing resource demand in combination with climate change-induced changes in resource supply. Quantifying the pools and fluxes constituting the terrestrial African carbon cycle is a challenge, because of uncertainties in meteorological driver data, lack of validation data, and potentially uncertain representation of important processes in major ecosystems. In this paper, terrestrial primary production estimates derived from remote sensing and a dynamic vegetation model are compared and quantified for major African land cover types.

**Results:**

Continental gross primary production estimates derived from remote sensing were higher than corresponding estimates derived from a dynamic vegetation model. However, estimates of continental net primary production from remote sensing were lower than corresponding estimates from the dynamic vegetation model. Variation was found among land cover classes, and the largest differences in gross primary production were found in the evergreen broadleaf forest. Average carbon use efficiency (NPP/GPP) was 0.58 for the vegetation model and 0.46 for the remote sensing method. Validation versus in situ data of aboveground net primary production revealed significant positive relationships for both methods. A combination of the remote sensing method with the dynamic vegetation model did not strongly affect this relationship.

**Conclusion:**

Observed significant differences in estimated vegetation productivity may have several causes, including model design and temperature sensitivity. Differences in carbon use efficiency reflect underlying model assumptions. Integrating the realistic process representation of dynamic vegetation models with the high resolution observational strength of remote sensing may support realistic estimation of components of the carbon cycle and enhance resource monitoring, providing suitable validation data is available.

## Background

Estimates of photosynthetic assimilation and respiration of carbon (C), along with fluxes from fires and other ecosystem disturbances, form the basis for quantifying the terrestrial carbon balance. Carbon balance studies and the understanding of factors controlling carbon fluxes, as well as their spatial and temporal variation, are key features of recent research relating to climate change [1-5].

Gross primary production (GPP) is the capacity of the vegetation to capture carbon and energy during photosynthesis. Net primary production (NPP) is the net carbon stored after subtracting the autotrophic plant respiration (Ra) from GPP. Ra is commonly divided into growth respiration, often assumed to be a fixed proportion of NPP, and maintenance respiration, which is dependent on temperature [6] and nitrogen content [7]. The influence of water availability and soil moisture (in addition to temperature) on heterotrophic respiration (Rh) in drier areas is well known [8,9], but recently a drought-induced decline in Ra has been shown for trees in Amazonas [10].

While some of the annual NPP in an ecosystem may be lost by episodic events like fire, the remainder constitutes essential ecosystem services [11,12] such as fuel, food, feed, fiber and construction materials [13]. As human access to these resources and services is crucial, monitoring of primary production is important in assessing the variability of resource availability and in evaluating the potential impact of climate change on plant production [14,15] and resource availability [16].

Recent work on the carbon budget of Africa reports that the continent is a small sink of carbon (−0.61 ± 0.58 Pg C yr^−1^) (1 Pg = 10^15^ g) [17] and stresses the importance of Africa in the global carbon cycle, despite considerable uncertainty. African ecosystems contribute about 20% of global NPP, 20% of heterotrophic respiration [18] and 20% of global land use CO_2_ emissions [19]. The atmospheric input from fossil fuel in Africa is low [20], but fire emissions are estimated to form about 40% of the global total, mostly from savanna burning [19,21]. Ciais *et al.* [19] used model analysis to report that GPP was more important than total ecosystem respiration (TER) in determining African net biome productivity.

Annual variability is larger for GPP than for TER and is mainly driven by rainfall [19,22], whereas TER is more dependent on temperature [23]. Jung *et al.* reported a mean GPP for Africa of 24.3 Pg ±2.9 C yr^−1^ for the period 1982–2008 [24]. Valentini *et al.*recently reported significant variability (for the period 1990–2009) in both GPP, ranging from 20.61 to 40.91 [Pg C yr^−1^] with a mean of 28.16, and NPP, ranging from 9.25 to 20.46 [Pg C yr^−1^] with a mean of 13.27 [17]. Much of the interannual variability of the global carbon cycle can be derived from the African continent [25], so it is important to quantify these fluxes from a carbon budget perspective as well as from a resource mapping perspective. Recent studies of potential climate change implications also stress the importance of related studies in Africa [17,19,25,26].

Remote sensing-based models, applying the concept of light use efficiency (LUE), and dynamic vegetation models (DVM) are two common approaches for assessing carbon budgets and for monitoring resources. Both methodologies can provide spatially and temporally distributed measures of GPP, NPP and Ra.

LUE-based models often assume a relatively constant assimilation rate of carbon per unit absorbed photosynthetically active radiation [27,28]. This rate is called the light use efficiency (ε) and is commonly expressed in g C MJ^−1^ APAR (absorbed photosynthetic active radiation). APAR is estimated as the product of incoming PAR and the fraction of absorbed PAR (FAPAR). FAPAR is derived from earth observation data, often with a spatial resolution of around 250–1000 m and weekly to monthly temporal resolution, from sensors such as the Advance Very High Resolution Radiometer (AVHRR), Moderate Resolution Imaging Spectrometer (MODIS) and the Satellite Pour l’Observation de la Terre (SPOT).

Vegetation indices such as the Normalized Difference Vegetation Index (NDVI) and the Enhanced Vegetation Index (EVI) are used to describe and quantify FAPAR, as they do this well [29,30]. Additional environmental constraints on assimilation, such as temperature or water availability (e.g. through soil moisture content, evaporative fraction or vapor pressure deficit), are usually estimated from meteorological data, but may also be derived directly from earth observation data [31]. This reduces dependency on data sources with comparatively low spatial resolution and potential delays in data availability through the need to process data originating from in situ measurements and meteorological reanalysis data sets. Recently suggested developments of LUE models include improved estimation of Ra, incorporation of diffuse radiation [4] and the use of variable LUE based on plant functional types or photosynthetic pathways [28]. LUE may also be determined directly using remote sensing, with a potential to provide improved estimates of the spatial variability of LUE compared to current methods [32,33].

DVMs integrate research from several disciplines, including plant geography, vegetation dynamics, biogeochemistry, plant physiology and biophysics [34]. They often present a more detailed representation of essential processes (photosynthesis, Ra, allocation of carbon, hydrology, etc.) and ecology (population dynamics, mortality, disturbances) when compared to LUE models, but are computationally more demanding. Driven by meteorology, CO_2_ concentration and soil data, often with low spatial resolution, and governed by plant functional types, DVMs can be used both as a diagnostic and a prognostic tool, allowing estimates of future responses to climatic change and thereby providing valuable information on forthcoming resource availability, as well as prognoses of future carbon budgets [35]. A wide range of DVMs are available; for an overview see [34,36].

The combination of ecological process models and satellite-derived information can involve several strategies. Plummer [37] identified a remote sensing-based generation of “*spatially comprehensive and temporally repeatable global data sets for use by ecological modellers*” as one of the more central. Verstraeten *et al.* [38] integrated the C-FIX model with soil moisture data derived from an active microwave sensor, and found that soil moisture had an impact on both magnitude and spatial pattern of carbon exchange fluxes. Smith *et al*. [39] investigated the feasibility of two methods (satellite-based estimates of FPAR and stand structure descriptions) to constrain dynamic ecosystem model behavior using data from remote sensing, and concluded the combination to *“…offer a promise as a step towards the development of operational tools…”*.

This study compares and combines the LPJ-GUESS dynamic vegetation model with the earth observation-based LUE model MOD17, both described in more detail below.

**Figure 1:**
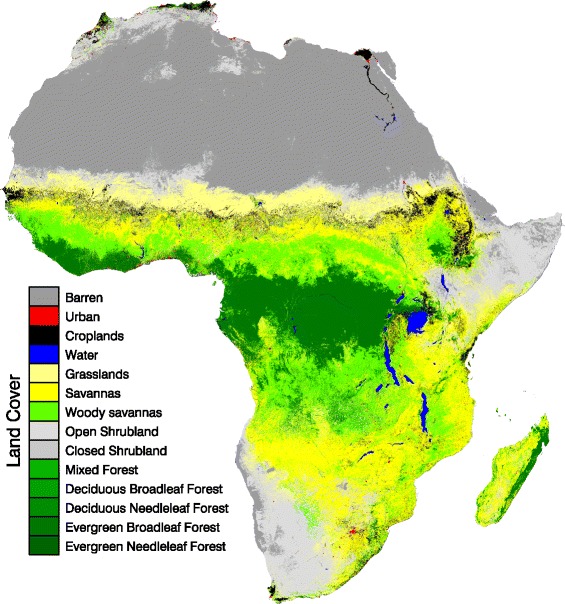
**Study area.** Land cover of Africa from MODIS (MOD12Q1). This data was used for looking up the correct biome parameters used in MOD17 from the Biome Property Look-Up Table (BPLUT), and describes the land cover classes used in analysis and their spatial distribution.

The differences between DVMs and LUE models in terms of driver data and utilized concepts can result in differences in estimated vegetation productivity. This study aims to describe and quantify the differences in estimated vegetation productivity for Africa (Figure 1), and also to combine the two methodologies using a simple approach. The study is divided into three tasks:

- Comparing GPP and NPP from MOD17 and LPJ-GUESS (Null hypothesis 1: MOD17 vegetation productivity = LPJ-GUESS vegetation productivity).
- Simple integration of the two methodologies (Null hypothesis 2: NPP estimated by LPJ-GUESS = NPP estimated by the combined approach.)
- Validation versus in situ measurements of vegetation productivity (Null hypothesis 3: Model estimated vegetation productivity = in situ measured vegetation productivity).

Can knowledge that promotes reliable methods for estimation of GPP and NPP in Africa be acquired through comparison and integration of LPJ-GUESS and MOD17? Does the combination of the observational strength of high spatial and temporal resolution earth observation data with the more realistic process representation of the DVM improve estimates of net primary productivity? The central findings of this study, produced while trying to answer the questions above, included general agreement regarding estimated vegetation productivity for most land cover classes except for the evergreen broadleaf forest, a moderate relationship when comparing model-estimated vegetation productivity versus in situ data, and potential benefits through a simple integration of the two model approaches.

## Results

### GPP and NPP for Africa

**Figure 2:**
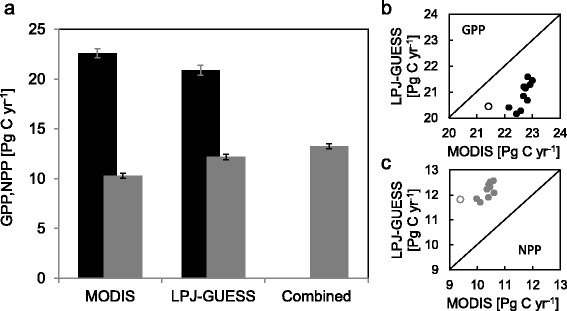
**GPP and NPP for Africa. (a)** mean GPP (black) and NPP (grey) from MOD17 and from the LPJ-GUESS model (average for the period 2000–2010), and mean NPP_Combined_. Error bars illustrate ± standard deviation. GPP and NPP differ significantly (LPJ-GUESS vs MOD17, t-test, p < 0.01), **(b)** Annual (2000–2010) MOD17 and LPJ-GUESS GPP [Pg C] and **(c)** NPP [Pg C], the open circle in b and c denote year 2005.

Mean annual GPP (2000–2010) for Africa was 22.6 ± 0.45 (±1 standard deviation) Pg (range 21.4-23.0) for MOD17 versus 20.9 ± 0.50 Pg for LPJ-GUESS (range 20.2-21.6) (Figure 2a). Mean annual NPP (2000–2010) for Africa was 10.3 ± 0.35 Pg for MOD17 (range 9.4-10.6) versus 12.2 ± 0.31 Pg for LPJ-GUESS (range 11.7-12-6) (Figure 2a). Total NPP_Combined_ was 13.3 Pg. There was a weak linear relationship (r^2^ = 0.39) between the annual sums of GPP for MOD17 and LPJ-GUESS (Figure 2b); removing the outlier year 2005 increased r^2^ to 0.55. A similar relationship was found for NPP (r^2^ = 0.43) (Figure 2c). MOD17 GPP was systematically higher than LPJ-GUESS GPP, but the opposite applied for NPP.

**Figure 3:**
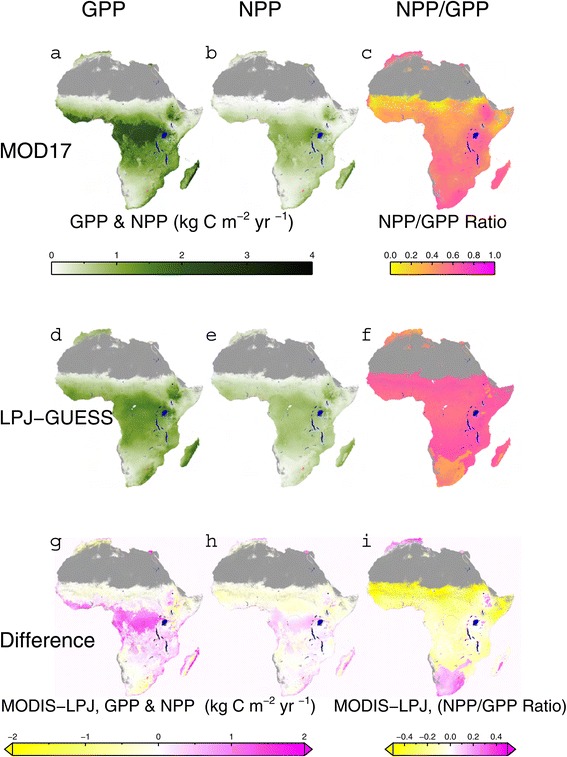
**GPP, NPP and the NPP/GPP ratio for MOD17 and for LPJ-GUESS.** Mean (2000–2010) **(a)** GPP and **(b)** NPP (both in kg C m^−2^ yr^−1^) for MOD17 and LPJ-GUESS **(d, e)**. Mean (2000–2010) NPP/GPP ratio for MOD17 **(c)** and LPJ-GUESS **(f)**. Water is blue, urban areas are black, and barren areas are grey). Difference (MOD17-LPJ GUESS) in GPP **(g)**, NPP **(h)** and NPP/GPP ratio **(i)**.

**Figure 4:**
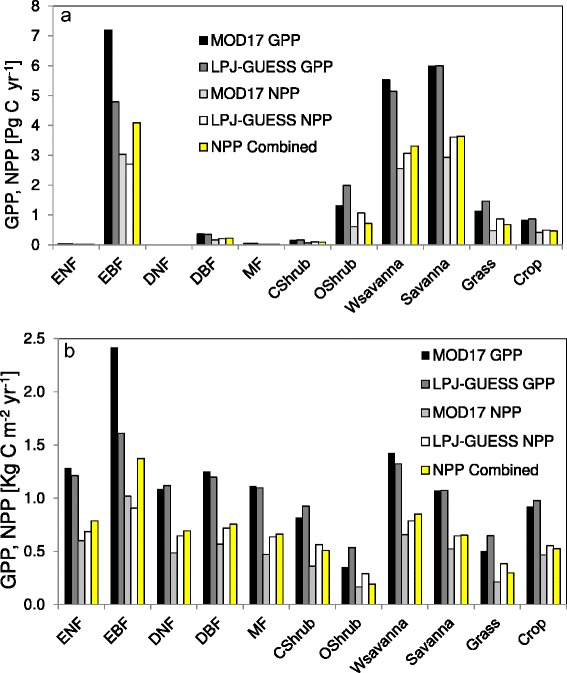
**GPP and NPP per land cover class. (a) **Totaled MOD17 and LPJ-GUESS GPP and NPP per land cover class, where GPP differs significantly (t-test p < 0.01) for all land cover types except for mixed forest and savannas. NPP differs significantly (t-test p < 0.01) for all land cover types. **(b)** Mean GPP and NPP per land cover class and unit area. Yellow bars denote NPP_Combined_ (MOD17 GPP – Ra from LPJ-GUESS).

The spatial pattern of NPP and GPP were similar for both methods but with some notable exceptions (Figure 3). Mean MOD17 GPP (2.41 kg C m^−2^ yr^−1^) was, on average, 0.8 kg C m^−2^ yr^−1^ higher (t-test, p < 0.01) than LPJ-GUESS GPP (1.61 kg C m^−2^ yr^−1^) for the evergreen broadleaf forest (EBF) (Figure 4a). This is equivalent to a total GPP for MOD17 exceeding LPJ-GUESS GPP by 2.4 Pg yr^−1^ for the EBF (Figure 4a). GPP of remaining forest types (DBF, DNF, ENF) differed less (<0.07 kg C m^−2^ yr^−1^) between MOD17 and LPJ-GUESS, and ranged from 1.09 to 1.29 kg C m^−2^ yr^−1^ for MOD17 and from 1.10 to 1.21 kg C m^−2^ yr^−1^ for LPJ-GUESS (Figure 4b). These remaining forest types (DBF, DNF, EBF) have low areal extent and contribute only 2% (0.46 Pg) and 2.1% (0.44 Pg) to total GPP, for MOD17 and LPJ-GUESS, respectively (Figure 4a).

Average (2000–2010) GPP for remaining land cover classes (Wsavanna, Savanna OShrub, CShrub and Grass) differed by less than 0.2 kg C m^−2^ yr^−1^ (Figure 4b), between MOD17 and LPJ-GUESS. These land cover classes, all with large areal extent, contribute a large proportion of total continental GPP (63% for MOD17 and 71% for LPJ-GUESS). Woody savannas have the second highest GPP per area after the evergreen broadleaf forest (2.41 kg C m^−2^ yr^−1^), averaging 1.42 kg C m^−2^ yr^−1^for MOD17 and 1.32 kg C m^−2^ yr^−1^ for LPJ-GUESS.

**Figure 5:**
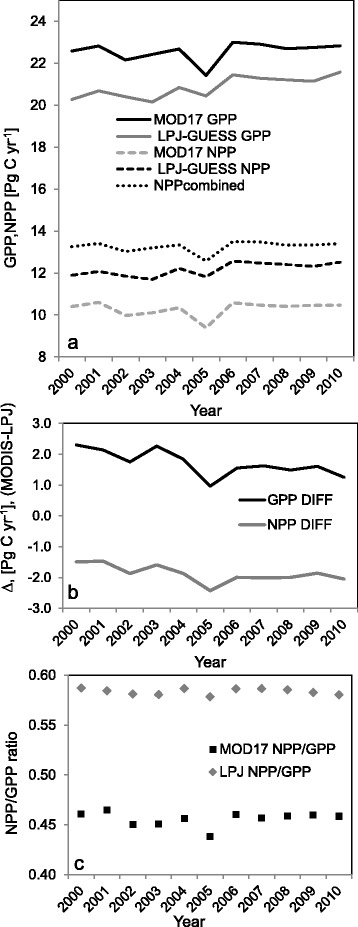
**Time series of vegetation productivity. (a)** Annual GPP and NPP for MOD17, LPJ-GUESS and the combined approach for the period 2000–2010, **(b)** differences (MOD17-LPJ-GUESS) and **(c)** the NPP/GPP ratio.

The temporal variability of each model is smaller than the differences between the models; both show a dip in 2005, but this is larger for MOD17 (Figure 5a). On average, MOD17 GPP for Africa was 1.7 Pg yr^−1^ higher than LPJ-GUESS GPP, while average MOD17 NPP was 1.9 Pg yr^−1^ lower than average LPJ-GUESS NPP. These differences were smallest for GPP (0.97 Pg) in 2005 and largest for NPP (−2.4 Pg) in 2005 (Figure 5b).

The average annual (2000–2010) NPP/GPP ratio was 0.456 (range 0.438-0.465) for MOD17 and 0.583 (range 0.578-0.587) for LPJ-GUESS (Figure 5c). For MOD17 the ratio among the land cover classes ranged from 0.35 (Grasslands) to 0.47 (Savannas), and for LPJ-GUESS from 0.55 (Woody Savanna) to 0.62 (Savannas). The NPP/GPP ratio for EBF was 0.42 for MOD17 and 0.56 for LPJ-GUESS. The temporal variability of the NPP/GPP ratio was small, especially for LPJ-GUESS and the lowest ratio for both methods occurred in 2005 (Figure 5c). Parts of southern Africa have a distinctly lower NPP/GPP ratio for LPJ-GUESS (Figure 3f). On average MOD17 assumed Ra to be about 79% (0.46/0.58) of LPJ-GUESS Ra.

### MOD17 quality control (QC) data

**Figure 6:**
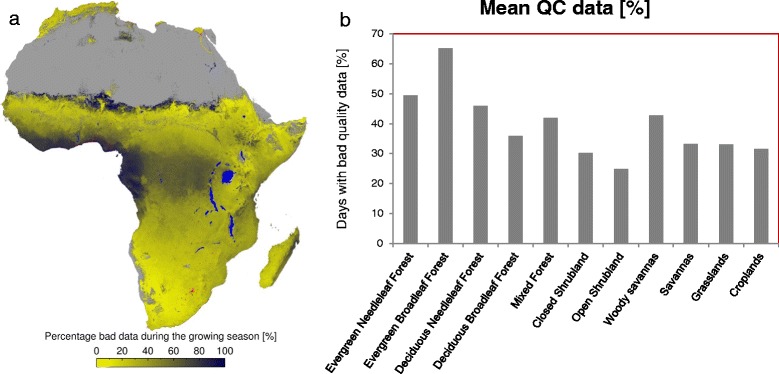
**MODIS quality control data. (a)** MOD17 QC data describing the percentage of days during the growing season with poor-quality data [%, average for 2000–2010] for Africa, **(b)** QC per land cover class.

QC values quantify the proportion of growing days (%) during the growing season that use artificially filled FPAR&LAI, so higher QC values = lower quality. High QC values are reached in the coastal areas of western Africa (QC >70%), in the Congo basin, and along a narrow band just south of the Sahara Desert (Figure 6), due to high frequency of clouds and aerosols. Southern Africa and other drier regions with less cloud have lower QC (assuming more reliable annual GPP and NPP) compared to the tropical forest areas and parts of the Ethiopian Highlands. Average QC values per land cover classes are 24% (OShrub), 32% (Crop), 33% (Savanna and Grass), 43% (Wsavanna), 36-49% (all forest types except EBF) and 65% (EBF) (Figure 6). The overall mean QC when all land cover classes are included is 39.5%.

### Comparison between in situ-collected NPP and model-estimated NPP

**Figure 7:**
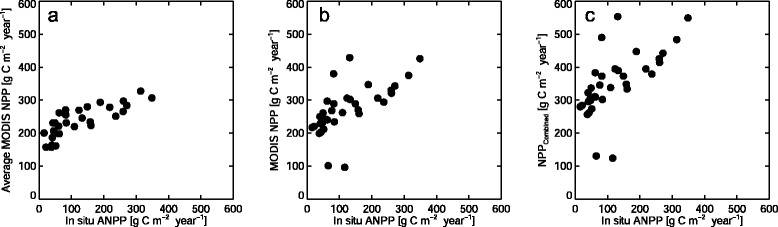
**Validation versus in situ data from Sudan.** NPP estimates versus field-based validation data of aboveground NPP (ANPP) from Sudan. **(a)** Average (2000–2010) MOD17 NPP (n = 31, r = 0.80, RMSE = 153 g), **(b)** MOD17 NPP for the same year (r = 0.64, RMSE = 145 g, n = 35), **(c)** NPP_Combined_ for the same year (r = 0.64, RMSE = 176 g, n = 35).

Average MOD17 NPP (2000–2010) is strongly correlated with in situ ANPP (r = 0.80, RMSE = 153 g) (Figure 7a). Using MOD17 NPP and in situ ANPP for Sudan collected the same year reduces the linear correlation (r = 0.64, RMSE = 145 g, Figure 7b). NPP_Combined_ are moderately correlated compared with in situ-collected ANPP (r = 0.64. RMSE = 176 g) for the same year. All MOD17 NPP estimates are systematically higher than the in situ data, which may partly be explained by the in situ data only representing aboveground biomass.

**Figure 8:**
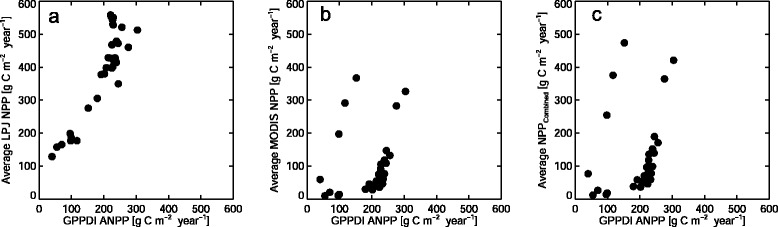
**Validation versus NPP Multi-Biome: Global Primary Production Data Initiative (GPPDI) above ground NPP (ANPP). (a)** Average (2000–2010) LPJ-GUESS NPP vs GPPDI (r = 0.90, RMSE =207 g), **(b)** Average (2000–2010) MOD17 NPP vs GPPDI (r = 0.21, RMSE =141 g) and **(c)** Average (2000–2010) NPP_Combined_ vs GPPDI (r = 0.21, RMSE = 142 g), n = 35.

The NPP Multi-biome data set [40] shows a strong linear relationship for LPJ-GUESS (r = 0.90, RMSE = 207 g) and a weaker linear relationship for MOD17 (r = 0.21, RMSE = 141 g) and NPP_Combined_ (Figure 8), using the 2000–2010 averages. Note that Figures 7 and 8 compare in situ collected aboveground NPP with total NPP (from MOD17 and LPJ-GUESS), and this influences the relationships illustrated.

**Figure 9:**
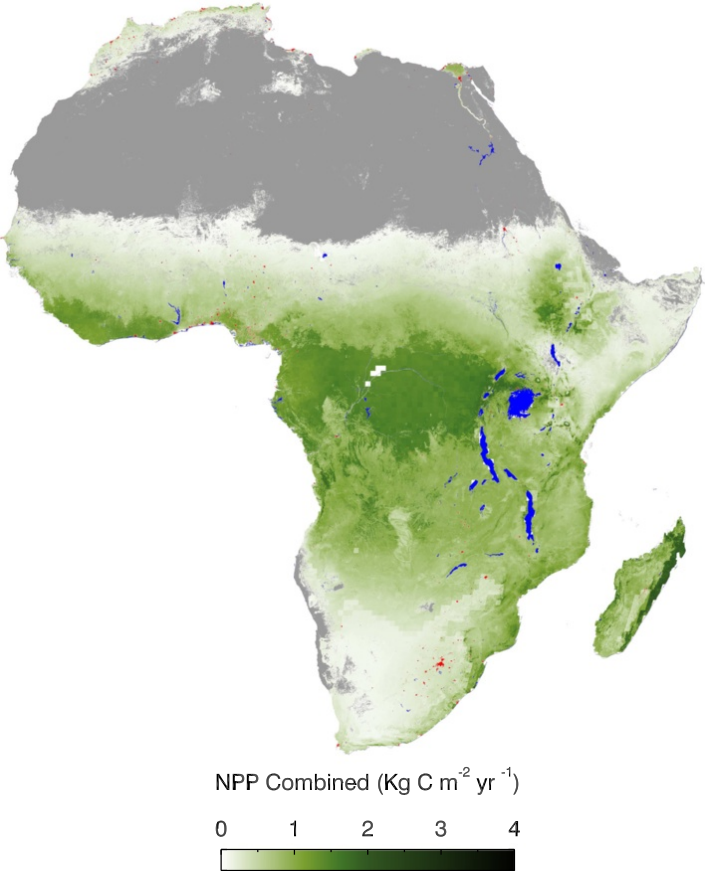
**Integrated NPP.** NPP_Combined_, produced by MOD17 GPP x NPP/GPP ratio from LPJ-GUESS, average for the 2000–2010 period.

**Figure 10:**
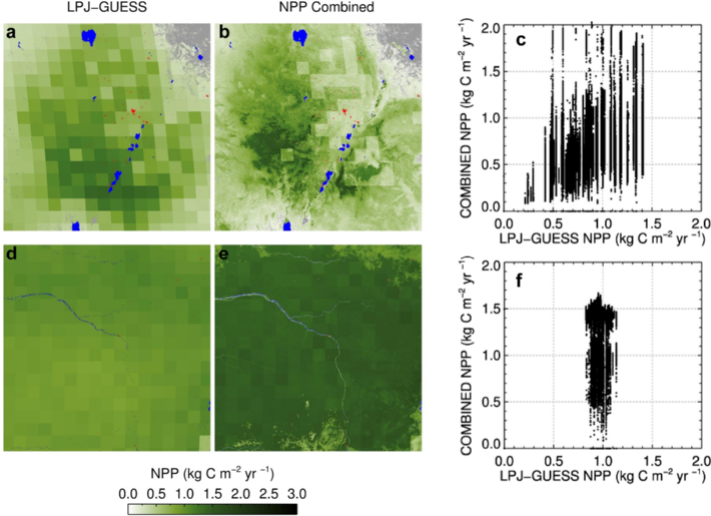
**Dynamic vegetation model NPP vs NPP**_Combined_ **.** NPP from **(a)** LPJ-GUESS and **(b)** NPP_Combined_ for a 1000 x 1000 km area in the Ethiopian Highlands and **(c)** a scatter plot of LPJ-GUESS NPP vs NPP_Combined._ **(d-f)** show the same as **(a-c)** bur for a 1000 x 1000 km are in the Congo Basin. Urban areas are red, water is blue, and barren areas are grey. NPP_Combined_ is calculated from MO17 GPP and LPJ-GUESS Ra.

NPP_Combined_ are illustrated in Figure 9, with a more detailed comparison with LPJ-GUESS NPP for areas centered on the Ethiopian Highlands and the Congo Basin (Figure 10). The effect of the increased spatial resolution (1 × 1 km) of MOD17 is visible, but the 0.5 × 0.5 degree pattern originating from LPJ-GUESS still remains for some regions. Figure 10c and f illustrate the relationship between LPJ-GUESS NPP and NPP_Combined_ for the two subareas.

## Discussion

Observed statistically significant differences in primary production between MOD17 and LPJ-GUESS support rejection of hypothesis 1 and exclude the possibility of both methods being fully correct (Figure 2). For the entire continent, MOD17 GPP exceeds LPJ-GUESS GPP by 1.7 Pg yr^−1^ on average for the study period (2000–2010). The differences are consistent over the study period (Figures 2 and 5) and can mainly be explained by the higher MOD17 GPP for the evergreen broadleaf forest (Figure 4). GPP of large land cover classes such as grasslands, savannas and woody savannas vary less. Both GPP and NPP reported here (Figure 2) are within the ranges shown in earlier results for Africa, even if the ranges are wide [17,24,41].

Average MOD17 NPP was 1.9 Pg yr^−1^ less than LPJ-GUESS NPP, which can largely be explained by a lower NPP/GPP ratio for MOD17, indicating substantially higher autotrophic respiration than LPJ-GUESS (Figures 3 and 5c). A similar result was reported from a recent study covering northern Eurasia, where a spatial correlation of 0.63 was found for mean annual GPP (2000–2009) derived from LPJG (a slightly modified version of LPJ-GUESS including methane from wetlands) versus MOD17 GPP [42]. LPJG explained about 40% of the variability found in the MOD17 product. Reported LPJG GPP was about 40% lower than MOD17 GPP, which may indicate that the LPJ-GUESS vs MOD17 GPP differences are not limited to Africa or warmer climates.

The higher proportion of GPP lost as autotrophic respiration by MOD17 has been attributed to an excessive temperature sensitivity of the MOD17 algorithm [43,44]. However, Clark *et al.* showed decreased increment in tropical trees due to high temperatures, and reported strong correlation between temperature-induced tree growth patterns and tropical CO_2_ fluxes. They suggested a “*remarkable sensitivity of the net carbon balance of tropical rain forests to increasing temperature”* [45], which is also supported by other studies [46]. Mahli *et al.* reported NPP ranging from 1.0 to 1.44 kg C m^−2^ yr^−1^, based on in situ measurements in Amazonian rainforests, close to the mean EBF NPP of MODIS (1.02 kg C m^−2^ yr^−1^) and LPJ-GUESS (0.91 kg C m^−2^ yr^−1^) found here (Figure 4b). Corresponding Amazonian carbon use efficiencies (NPP/GPP) ranged from 0.32 to 0.49 [47], overlapping well with the MOD17 NPP/GPP ratio of 0.42 found here for the evergreen broadleaf forest, but lower than the 0.56 of LPJ-GUESS.

For large areas of Africa, the NPP/GPP ratio for MOD17 was lower than for LPJ-GUESS (Figure 3), indicating a higher autotrophic respiration rate for MOD17. Southern Africa, Morocco & Northern Tunisia and parts of Ethiopia are exceptions to this, with lower NPP/GPP ratio for LPJ-GUESS.

In a global study, Ahlström reported that differences in meteorological forcing data used by LPJ-GUESS and MOD17 had small effects on estimated vegetation productivity [48]. However, Traore *et al.* suggested that the uncertainty in the ORCHIDEE model is strongly related to the meteorological forcing data in regions with sparse weather station data [49], something potentially affecting all models utilizing driver data derived from weather station data. From this study, no conclusions can be drawn on what determines the spatial pattern and observed differences in the NPP/GPP ratio among land cover classes, but effects of model formulation such as the temperature sensitivity and/or propertires of plant functional types (LPJ-GUESS) and biome specific parameters (MOD17) may be contributing factors.

Zhang *et al.* [50] reported a global average MOD17 NPP/GPP ratio of 0.52, higher than the results presented here for Africa (0.46). The MOD17 data used by Zhang *et al.* was driven by meteorological data from the NASA Data Assimilation Office (DAO) which is not fully comparable with later versions of MOD17 using NCEP climate driver data [50]. Amthor [51] suggested 0.35-0.80 as the ‘allowable’ range for the NPP/GPP ratio (with 0.45-0.60 as a more realistic alternative) and proposed that available data is not sufficient to decide how constrained the NPP/GPP ratio is across species and environments. Valentini *et al.* reported a NPP/GPP ratio ranging from 0.37 to 0.59 when comparing nine DGVMs, with a continental NPP for Africa ranging from 9.25 to 20.46 Pg C yr^−1^and a GPP ranging from 20.61 to 40.91 Pg C yr^−1^. This illustrates substantial uncertainty in vegetation productivity estimates for Africa, including both magnitudes and the carbon use efficiency (NPP/GPP) [17] in addition to the limited field data (eddy covariance measurements, long term ecological field experiments and similar) available for tropical and subtropical areas [52].

Assuming a global GPP of ~120 Pg, and applying a carbon use efficiency of 0.58 versus 0.46, yields a difference in estimated global NPP of 14.4 Pg (69.6-55.2). Even if these assumptions hardly are applicable globally, 14.4 Pg C ‘lost in modelling space’ is enough to encourage further investigations and additional collection of calibration & validation data and long-term ecosystem experimental data sets from tropical regions, such as Africa. Integration of different types of methods, such as remote sensing, data-oriented methods and process-oriented models, may help to produce consistent estimates of primary productivity and respired fraction, and increase knowledge about involved ecosystem processes and their drivers.

Validation of model estimates using in situ measurements provides vital information on model performance [6,53,54], even if scale differences, sampling strategies and methodological discrepancies in data collection reduce comparability. Significant positive relationships between estimated NPP versus field data were found for both models (Figures 7 and 8), indicating a general applicability of both models, as shown earlier for MOD17 [54,55] and LPJ-GUESS [56,57]. The significant positive relationships of estimated NPP versus in situ collected NPP suggest that hypothesis 3 cannot be rejected (Figures 7 and 8). A stronger linear relationship between in situ NPP data and model estimates favors LPJ-GUESS (Figure 8) whereas a lower RMSE and no systematic overestimation favor MOD17. Bias and differences arising from comparing NPP with ANPP introduce additional uncertainty, and no definite conclusion can be drawn from the current small data set available, except general significant positive relationships (Figures 7 and 8). The limited spatial distribution of the validation data (Senegal and semi-arid Sudan) limited the representativity, as major biomes such as the evergreen broadleaf forest are missing. A coordinated effort to compile continent-wide in situ data for calibration and validation of methods estimating biomass and NPP could enhance the possibilities for statistically sound and representative evaluation of methods estimating vegetation productivity for Africa.

Integrating MOD17 and LPJ-GUESS by applying the NPP/GPP ratio from the dynamic vegetation model to the observational and high spatial resolution remote sensing model GPP seems reasonable (Figure 10) if we assume a proper process representation in LPJ-GUESS. However, the validation does not support any clear improvement (Figures 7 and 8), even if estimated NPP of MOD17, LPJ-GUESS and NPP_Combined_ differ on a continental scale (Figure 2) and for individual land cover classes (Figure 4). There is also a risk of violation of internal model logic due to differences in model assumptions and formulations when applying the LPJ-GUESS NPP/GPP ratio to MOD17 GPP. Consequently, there is support both for and against rejection of hypothesis 2.

The temporal variability of each method is smaller than the differences between the models. The dry year 2005 [15] yields a clear dip for MOD17 GPP but only a small dip for LPJ-GUESS GPP, whereas the lowest NPP/GPP ratio for both models occurs in 2005 (Figure 5), supporting increased respiration during dry conditions [58]. The NPP/GPP ratio in 2005 for MOD17 was 0.438 versus 0.578 for LPJ-GUESS, a deviation of 3.8% and 0.9% respectively from the 2000–2010 average NPP/GPP ratio. This indicates that MOD17 responds more strongly to the drought in 2005, potentially through higher temperature. In general, the temporal variability is similar, indicating similar forcing and similar response to precipitation and moisture availability, both important drivers of vegetation productivity in drier parts of Africa [22,59].

The evergreen broadleaf forest has the lowest satellite data reliability (Figure 6) among all land cover classes, partly due to frequent cloud cover [60]. The large proportion (QC = 65%) of FPAR data originating from MOD15 and used in MOD17 that is replaced by interpolated values may impact the GPP estimation [54] and increase the uncertainty of estimated GPP. Areas affected by frequent cloud cover, such as parts of the evergreen broadleaf forest, may then show less reliable estimates of vegetation productivity using MOD17 (or any method based on optical remote sensing). All land cover classes are affected by this, as most areas have QC values > 30%, and the overall mean is 39.5%, indicating a need for effective gap-filling methods [54,61].

Estimated global median GPP for the tropical forest, by Beer *et al.* [59] of 2.34 kg C m^−2^ yr^−1^ (median of seven different up-scaling schemes) is close to the 2.41 kg C m^−2^ yr^−1^estimated by MOD17 (for EBF) but clearly exceeds the 1.61 kg C m^−2^ yr^−1^ estimated by LPJ-GUESS (Figures 3 and 4). Fisher *et al.* [62] estimated average GPP of the African tropical forest to range from about 1.4 to 4.0 kg C m^−2^ yr^−1^, indicating large variability among applied global dynamic vegetation models. Without in situ validation data, the absolute magnitudes of plant productivity is difficult to evaluate, and focus is often on interannual variability related to climatic drivers [41,62].

The higher spatial resolution gained through using observational data such as MOD17 enables estimates of driver variables such as temperature, vapor pressure deficit [63] and incoming PAR [64] with high spatial and temporal resolution. This may reduce dependency on climate data sets like NCEP and CRU, facilitating high resolution estimates of vegetation productivity, especially in areas with low density of meteorological observations [62,65]. Using observational data on spatial distribution of GPP and NPP within LPJ-GUESS grid cells could increase spatial information content while keeping LPJ-GUESS within grid cell integrity (e.g. sums of GPP and NPP). From a local to regional resource monitoring perspective [16], earth observation provides additional useful spatial and temporal resolution (Figure 10), while regions with frequent cloud cover reduce the possibilities for robust continent-wide monitoring of plant productivity in Africa.

## Conclusion

The results from this study suggest significant differences in (1.7 Pg C yr^−1^) between estimated continental GPP from MOD17 and from LPJ-GUESS, mainly originating from higher MOD17 GPP for the evergreen broadleaf forest biome. The causes of this difference are not determined, but reduced availability of cloud-free earth observation data may cause uncertainties in the MOD17 estimates.

Substantial differences in carbon use efficiency (NPP/GPP ratio) result in continental NPP from LPJ-GUESS exceeding (1.9 Pg C yr^−1^) NPP from MOD17, even if extensive land cover classes such as Savannas and Cropland show reasonable similarities in estimated GPP and NPP produced by both models.

Merging MOD17 GPP with the NPP/GPP ratio from LPJ-GUESS could help combine the high spatial resolution of the remote sensing-based MOD17 model with the more process-oriented LPJ-GUESS model. Utilization of preferred elements originating from different modelling schemes or representing different spatial & temporal regions or resolution may be beneficial in resource assessment and carbon cycle studies.

Unfortunately, as long as availability and accessibility of local meteorological data and in situ validation data (eddy covariance measurements, long-term ecological field experiments and similar) remain low, we can expect the quality and representativity of vegetation productivity estimates for Africa to remain hard to determine. Based on current available data may both methods be considered to produce plausible estimates of vegetation productivity for Africa.

## Methods

### Study area

This study covers Africa (Figure 1) with an area of approximately 30 million km^2^, including savannas (Savanna, 5.6 million km^2^), woody savannas (WSavanna, 3.9 million km^2^), open shrublands (OShrub, 3.7 million km^2^) evergreen broadleaf forest (EBF, 3.0 million km^2^), grassland (Grass, 2.2 million km^2^), croplands (Crop, 0.89 million km^2^), and closed shrublands (CShrub, 0.18 million km^2^). Other forest types (Evergreen Needleleaf Forest (ENF), Deciduous Needleleaf Forest (DLF), Deciduous Broadleaf Forest (DBF) and Mixed Forest (MF)) cover a total of 0.37 million km^2^. Other land cover classes, not considered here as MOD17 does not calculate GPP and NPP for them, include barren and sparsely vegetated areas (9.8 million km^2^), water (0.25 million km^2^), and urban & built up areas (53600 km^2^) (Figure 1).

### LPJ-GUESS

LPJ-GUESS is an “object oriented, modular framework for modelling the dynamics of ecosystem structure and functioning at scales from the patch to the globe, and at varying levels of process detail” [66]. The framework incorporates process-based representations of plant physiology and ecosystem biogeochemistry. The model is driven by atmospheric CO_2_ concentration, temperature, precipitation, radiation, and soil physical properties. Photosynthesis, respiration, stomatal conductance and phenology are simulated using a daily time step. Eleven plant functional types are used to represent vegetation [48]. Model output includes GPP, NPP, respiration, carbon pools and potential vegetation among a range of other possible outputs. LPJ-GUESS performs well when compared to other vegetation models [56] and predicts present day GPP in agreement with observation-based estimates [57]. Details on the LPJ-GUESS model are available in [6,67].

Annual GPP and NPP (kg C m^−2^ year^−1^), estimated by LPJ-GUESS and with a spatial resolution of 0.5 × 0.5 degrees (longitude, latitude), for the period 2000–2010 were used [48]. The model was forced with monthly precipitation, number of rain-days (days with >1 mm precipitation), incoming short-wave radiation and temperature from the CRU-NCEP (v2.0) dataset. Annual CO_2_ concentration was set from atmospheric and ice core measurements as described in [48].

GPP and NPP were resampled to the MOD17 grid (1 km spatial resolution and an equal area Sinusoidal projection). Grid cells without valid MOD17 GPP and NPP (MOD12Q1 land cover barren, water, or urban, Figure 1) were masked out from the LPJ-GUESS data in order to make the data sets comparable (i.e. identical spatial extent, land cover classes and number of grid cells).

### MOD17

MOD17 is based on the LUE concept and consists of two products, MOD17A2 and MOD17A3. MOD17A2 contains both 8-day GPP and 8-day net photosynthesis (PSNnet), whereas MOD17A3 contains annual sums of GPP, NPP and quality (QC) data [15,68].

The MOD17 algorithm calculates daily GPP as:

$$ \mathrm{G}\mathrm{P}\mathrm{P}={\varepsilon}_{\max}\times 0.45\times S{W}_{rad}\times \mathrm{FAPAR}\times f\left(\mathrm{V}\mathrm{P}\mathrm{D}\right)\times f\left({T}_{\min}\right) $$

where ε_max_ is the maximal, biome-specific light use efficiency [g C MJ^−1^], SW*rad* is incoming short-wave radiation (assuming 45% to be PAR), FAPAR is the fraction of absorbed PAR, f(VPD) and f(T_min_) are linear scalars reducing GPP due to water and temperature stress.

MOD17 GPP uses National Center for Environmental Prediction-Department of Energy (NCEP-DOE) reanalysis II data for Tmin, VPD and SWrad [69,70]. The NCEP-DOE reanalysis II data is interpolated from the original resolution of approximately 1.9° latitude × 1.9° longitude to 1 × 1 km grid cells [60]. FAPAR in the MOD17 GPP algorithm is derived from the 8-day MOD15A2 1 km product, and the 1 km University of Maryland (UMD) land cover classification scheme in the MOD12Q1 product (Figure 1) is used to map biome-specific physiological parameters (ε_max_, minimum temperature, VPD etc.) from the Biome Property Look-Up Table (BPLUT). NPP is calculated annually:

$$ NPP={\displaystyle \sum_{i=1}^{365} PsnNet-\left({R}_{mo}+{R}_g\right),} $$

where PsnNet = GPP-R_ml_-R_mr_. The maintenance respiration by leaves (R_ml_) and fine roots (R_mr_) respectively are calculated daily. R_mo_ is the annual maintenance respiration by all other living parts except leaves and fine roots, R_g_ is the annual growth respiration.

A recent evaluation of MOD17 GPP for Africa, based on eddy covariance data, concluded that MOD17A2 underestimated GPP by a mean difference of 0.70 g C m^−2^ day^−1^ but seasonality was captured well [54]. The underestimation was larger for drier sites, and differences between driver data (climatology from NCEP/DOE II and FAPAR from MOD15) and field observations of the same variables were found. Several studies have pointed out problems at drier sites [71-73] and suggest that prescribed MOD17A2 ε_max_ causes underestimation of MOD17A2 GPP [54,71,73]. Plummer concluded that the global performance of the MOD17 GPP is good under unstressed conditions, but suggested adaption of ε_max_ to better account for spatial and temporal within-biome variation [74]. MOD17 PsnNet and MOD17 NPP have also been reported to underestimate NPP when compared to field-measured aboveground NPP in Senegal [75].

MOD17A3 (UM Collection 5) annual totals of GPP and NPP for the years 2000–2010 were downloaded from ftp.ntsg.umt.edu and mosaicked for Africa at a 1 × 1 km spatial resolution and an equal area sinusoidal projection. Land cover (MOD12Q1) used by the BPLUT in MOD17 (same resolution and projection as MOD17A3) were downloaded from ftp://ftp.ntsg.umt.edu/.autofs/NTSG_Products/MOD12Q1_FOR_MOD15-17 and used for land cover stratification (Figure 1).

### Simple integration

The mean LPJ-GUESS NPP/GPP ratio for the 2000–2010 period was calculated (average NPP_(2000–2010)_/average GPP_(2000–2010)_) for each 1 × 1 km grid cell and multiplied by the 1 × 1 km MOD17 GPP, resulting in a 1 × 1 km spatial resolution NPP dataset, denoted NPP_Combined_ *.* This simple combination thereby applies the autotrophic respiration calculated from LPJ-GUESS to MOD17 GPP. The ratio of NPP to GPP is sometimes referred to as carbon use efficiency, and it is often assumed to have a constant value ~ 0.5 [76].

### MOD17 QC data

A MOD17 quality control (QC) measure is used to quantify the proportion of growing days (%) during the growing season that use artificially filled FPAR/LAI (originating from the MOD15A2 FAPAR/LAI product) due to cloud cover, to calculate 8-GPP and annual GPP and NPP.

$$ \mathrm{Q}\mathrm{C}=\left(\mathrm{N}\mathrm{U}\mathrm{g}/\mathrm{TOTALg}\right)\times 100 $$

where NUg is the number of days during the growing season with unreliable or missing MOD15 LAI inputs, and TOTALg is the total number of days in the growing season [60]. The growing season is defined as all days with Tmin above −8°C, also used as the minimum temperature control on photosynthesis for all biomes [60]. This temperature is only occasionally reached in Africa, and then mainly at higher elevations, so TOTALg = 365 in most cases. The QC data of MOD17A2 is inherited from the MOD15A2 FAPAR/LAI product and totaled to an annual value in MOD17A3 [60]. Average (2000–2010) QC for each land cover class was calculated.

### Validation using in situ data

Scale differences and temporal sampling make evaluation of coarse to moderate spatial resolution remote sensing models and DVMs difficult using in situ measurements of NPP, as they represent distinctly smaller areas. Despite this, two field-based data sets are used for comparison with model estimates to permit validation.

Firstly, 35 sites were available in Sudan with in situ collected aboveground NPP data [77]. The sampling sites were distributed along a north to south precipitation gradient in Northern Kordofan, from approximately 200 mm yr^−1^ in the north to about 600 mm yr^−1^ in the south. Each site was an area of approximately 300 × 300 m, with homogeneous vegetation selected. The data was collected from 2008 to 2010, using nested quadrats with 10 × 10 m quadrats for tree biomass and 1 × 1 m quadrats for the field layer (herbaceous biomass). Aboveground NPP of the field layer was assumed to equal the dry weight of the green herbaceous biomass at the end of the vegetation season. Tree NPP was estimated using allometric equations [78]. On each site, 20 to 24 quadrats were sampled and on most sites only aboveground biomass data was collected; details in [77]. Biomass was assumed to contain 50% C. For each of these 35 sites, MOD17 NPP and NPP_Combined_ for the corresponding grid cells and corresponding sampling year were extracted and compared to the in situ collected data. Average MOD17 NPP for the period 2000 to 2010 was also calculated for each site. Four sites without MOD17 data available for all years in the 2000–2010 period were removed when in situ NPP was compared with average MOD17 NPP. Due to differences in size, and as this in situ NPP data only overlap six different LPJ-GUESS grid cells (0.5 x 0.5 degrees), this data was not compared to LPJ-GUESS NPP.

Secondly, LPJ-GUESS NPP, MOD17 NPP and NPP_Combined_ were compared to NPP data from the Global Primary Production Data Initiative [40,79]. This data was available for 32 0.5° × 0.5° cells in West Africa (Senegal) and constituted aboveground NPP (ANPP) representative of the 1987–1997 period. Even if the time period of data collection does not overlap, this data may be informative regarding the general relationship between model estimates versus in situ estimates of vegetation productivity.

## Abbreviations

NPP: Net primary production
GPP: Gross primary production
LPJ-GUESS: Lund Potsdam Jena - General EcoSystem Simulator
Ra: Autotrophic plant respiration
TER: Total ecosystem respiration
